# Volatile organic compounds in truffle (*Tuber magnatum* Pico): comparison of samples from different regions of Italy and from different seasons

**DOI:** 10.1038/srep12629

**Published:** 2015-07-30

**Authors:** Vita Federico, Taiti Cosimo, Pompeiano Antonio, Bazihizina Nadia, Lucarotti Valentina, Mancuso Stefano, Alpi Amedeo

**Affiliations:** 1 Department of Agriculture, Food and Environment, University of Pisa, Pisa, Italy; 2 LINV-Department of Plant Soil and Environmental Science, University of Florence, Florence, Italy; 3 Laboratory of Plant Physiology, Center of Agricultural Sciences, Federal University of Alagoas, Maceió, AL, Brazil; 4 A.R.E.A. Foundation, Pisa, Italy; 5 Department of Agraria, Mediterranea University of Reggio Calabria, Salita Melissari, 89124 Reggio Calabria, Italy; 6 Department of Agricultural and Forest Sciences, viale delle Scienze Ed., 4, 90128 Palermo, Italy

## Abstract

In this paper volatile organic compounds (VOCs) from *Tuber magnatum* fruiting bodies were analyzed using a PTR-TOF-MS instrument. The aim was to characterize the VOC's profile of the fruiting bodies and identify if any VOCs were specific to a season and geographical areas. Multiple factorial analysis (MFA) was carried out on the signals obtained by MS. Experiments using ITS region sequencing proved that the *T. magnatum* life cycle includes the formation of fruiting bodies at two different times of the year. The VOCs profiles diverge when different seasonal and geographical productions are considered. Using PTR-TOF-MS, compounds present at levels as low pptv were detected. This made it possible to determine both the origin of fruiting bodies (Alba and San Miniato) and the two biological phases of fruiting bodies formation in San Miniato truffles.

Fungi included in the genus *Tuber* spp. are ascomycetes belonging to Pezizales, a large group of ectomycorrhizal fungi growing in symbiosis with the roots of several vascular plant species belonging to both Angiosperms and Gymnosperms. The ascoma of this fungus is a hypogeous complex apothecium, commonly known as a truffle. The *Tuber* genus is one of the 5 genera which make up the Tuberaceae family (subphylum Pezizomycotina) and it is estimated to comprise at least 180 species worldwide. These mainly occur in the temperate zones of the northern hemisphere, with three main regions of genetic differentiation: Europe, South East Asia and North America. The truffles are valued for two important features: they bring benefits to the forest ecosystems and to the host plant, as a result of their colonization by the mycorrhizae, and some of the species are edible and have higher economic value^1^ than other food crops^2^.

The truffle life cycle, like that of other symbiotic filamentous fungi, begins with a limited extraradical phase of vegetative growth in which the hyphae proliferate before coming into contact with the roots of the host plant (phase 1–2). Once this contact (phase 3) is established, the symbiotic phase begins, leading to the development of the ectomycorrhiza (phase 4), a new organ which is functionally and morphologically distinct from the two original partners. In the final stage, the mycelium is organised into the fruit body (phase 5–6), the role of which is to produce sexual fructifications to be dispersed in the environment at a later date. Vegetative mycelia then develop from these fructifications, originating a new extraradical phase and completing the truffle life cycle. Fruiting bodies are normally collected during the fall/winter (October-December, phase 5–6). The mycelium may produce a further sexual fructification during the summer (June-August), commonly known in Italy as “Marcia” (same developmental phase but different season). Summer fruiting bodies are not described in literature but are well known by field experts^3^.

Truffle's fruiting bodies release a mixture of volatile compounds which are likely used to communicate with plants, animals and microorganisms^4^. Other than their biological function, the mixture of volatile compounds (aroma) emitted from the fruiting bodies determines their economic value. The most valued species on the food market are *Tuber magnatum* Pico or "white truffle” and the *Tuber melanosporum* Vittadini, or “black truffle”^5^. Of the two, the *T. magnatum* is the more expensive one^1^. This truffle species has limited geographical distribution. It grows in spontaneous colonies in some regions in Italy (Tuscany, Piedmont, Marche, Umbria), as well as in Istria and several Balkan regions^1^. The mycorrhizal symbiosis is based on a mutual exchange of resources: the fungus brings limiting nutrients to the relationship in return for organic carbon structures that it gets from the plant. The plants and fungi's symbiotic relationship is essential for the fungi in order to be able to complete their life cycle. Unless they form a symbiotic relationships with plant roots and establish ectomycorrhizas, truffles do not form fruiting bodies^4^. Bacteria are the third component of mycorrhizal associations. They also, as they are loosely or tightly associated with mycorrhizal fungi, are thought to play a role in mycorrhizal function^6^. Mycorrhizal colonization of the plant roots induces the so-called “mycorrhizosphere effect”, which seems to favour the occurrence of bacteria involved in the mycorrhizal process (mycorrhization helper bacteria, or MHB) and ectomycorrhiza-associated bacteria (EMAB). These bacteria complement the roles of the external mycelium by mobilizing nutrients from minerals^7^ or through the production of volatile organic compounds^8^ that could contribute to truffle aroma in association with other *Tuber*-associated microbes (yeast and other fungi)^9^.

Truffle aroma is very unique and the complex composition of its volatile compounds has been the object of a number of studies over the past 20 years, which have employed a variety of techniques^10^ each of which have focused on different goals. For example, one study analysed *T. magnatum* volatiles^11^, and compared them to those present in other truffle species^12^. In another, an attempt was made to link the VOCs profiles to the geographical origin of the truffles using samples collected from different regions in Italy^5^. A more recent study showed how the effect different post-harvest conditions can have on the quantity and quality of VOCs in *T. magnatum*^10^.

Analysis of truffle volatiles has mainly been done using gas chromatography-mass spectrometry (GC-MS) of volatiles concentrated using suitable techniques, e.g. dynamic headspace GC-MS and purge-and-trap GC-MS^11^,^13^.

In numerous publications, another technique known as "Headspace Solid-phase Microextraction (HS-SPME) coupled with GC-MS" has been employed as a way to better identify volatile compounds in several truffles species as shown in several papers^14^,^15^,^16^,^17^.

The benefits of using GC-MS based methods to detect volatiles have also been compared and contrasted with other analytical systems. One such system is the "Proton Transfer Reaction—Mass Spectrometer (PTR-MS)", a soft chemical ionization procedure that allows on-line measurements of trace components with concentrations as low as a few pptv (parts per trillion by volume). This technique constitutes a valid alternative to GC-based methods, as it makes possible fast, accurate and direct measurement of volatile organic compounds, in this case in *T. magnatum*^18^. Significant improvements have been made in PTR-MS technology based on time-of-flight (TOF-MS)^19^. PTR-TOF-MS instruments can generate entire mass spectra (snapshots) of complex trace gas mixtures in short response times with high mass resolution and with virtually no upper mass limit^19^. This technique is used in the field of food science and technology to obtain a rapid, direct and non-invasive readings of volatiles. For example, it has proved useful to differentiate between specialty coffees^20^, to identify markers of origin in various protected designation of origin (PDO) of Netherlands cheeses^21^, and to evaluate the influence of sugar composition on flavor release in a strawberry flavored cereal bar system^22^. Recently this technique has also been used successfully to rapidly determine the volatile compounds present in the fruits of *Capsicum* spp^23^.

Taking full advantage of these recent innovations in analytical systems, this paper presents the results of a study that compared the volatile organic compounds in *T. magnatum* Pico fruiting bodies gathered from natural colonies in the Tuscany region with the fruiting bodies from the Piedmont region (the two most economically valuable of the Italian sub-species). In addition, fruiting bodies from two different seasons, were both collected in Tuscany: summer fruiting bodies were compared with fruiting bodies picked during the winter. All specimens were analyzed using PTR-MS.

## Results

### Chemical composition of the Aroma

**Figure 1:**
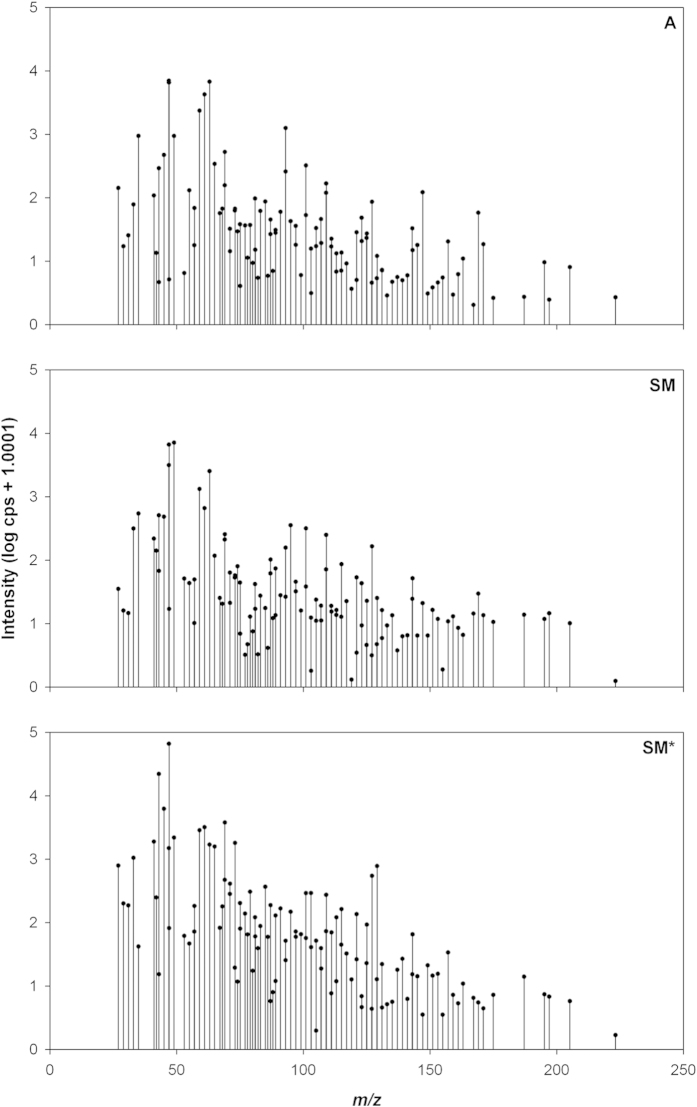
VOCs profile with their relative quantity in the three populations of samples coming from Tuscany (San Miniato winter - SM; San Miniato summer - SM*) and Piedmont (Alba - A). The graph shows the total area of the identified signals (x axis: signal intensities; y axis: m/z ratio) for each population.

Analysis of volatiles from *T. magnatum* fruiting bodies has led to the identification of 111 compounds (Table 1). The quantification of each compound is presented in Fig. 1. The 83% of the identified compounds were detected in the *m/z* range from 50 to 180 whereas the most abundant compounds were detected below *m/z* 50. The compounds identified are listed in Table 1 and classified on the base of their *m/z* ratio (both theoretical and measured), chemical name, molecular formula and the related literature. Citations were divided into four columns: one referring to previous PTR-MS data, the other three were respectively assigned to *T. magnatum*, other *Tuber* sp. and other fungi and bacteria. The profile of VOCs were similar for the three populations of samples with a general decrease in signal intensity due to the rise of the *m/z* ratio (Fig. 1).

To better describe the relationship between specific role of VOCs and the geographic origin as well as the harvesting seasons, the compounds were divided into 6 different chemical classes (Hydrocarbons, H; Aromatic hydrocarbons, AH; Phenols, P; Sulphur compounds, S; ; Terpenes, T; ,Others compounds, O), which could be treated as six groups of variables.

**Figure 2:**
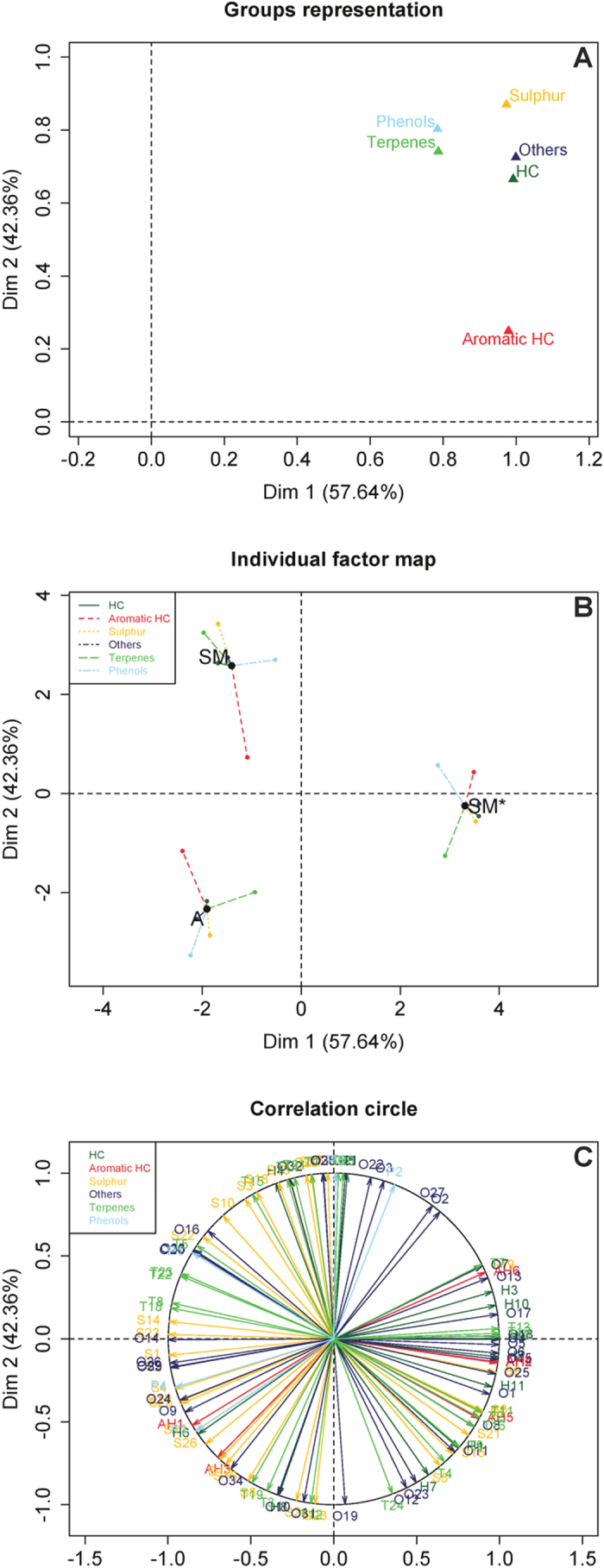
Multiple factor analysis (MFA) of transformed PTR-TOF-MS data for the 111 compounds identified in the 3 distinct populations of *Tuber magnatum*. (**A**) Representation of the groups of variables. Key: Darkgreen “HC", hydrocarbons; red “Aromatic HC”, aromatic hydrocarbons; golden “Sulphur”, sulphur compounds; midnightblue “Others”, others compounds; limegreen “Terpenes”, terpenes; lightskyblue “Phenols”, phenols, respectively; (**B**) Representation of the projection of variables onto the plane defined by the two first principal components of MFA. The coordinates of each variable are the correlation coefficients with the two first principal components. Key: SM = San Miniato Winter; A = Alba; SM* = San Miniato Summer, respectively; (**C**) Vector representation of the contribution of each compound to the distinction of the populations of samples. The coordinates of each variable are the correlation coefficients with the two first principal components. Key: Darkgreen “H”, 1–11 hydrocarbons; red “AH” 1–6, aromatic hydrocarbons; goldenrod “S”, 1–30 sulphur compounds; midnightblue “O”, 1–35 others compounds; limegreen “T”, 1–24 terpenes; lightskyblue “P”, 1–5 phenols, respectively.

MFA revealed the canonical relationship between the data obtained from PTR-TOF-MS fingerprints for the samples originating from the two geographical regions and different harvest times. The coordinates of the six group of variables were displayed and used to create a map of the group of compounds (Fig. 2a, Groups representation). The coordinates were calculated using the first two dimensions of the MFA (Dim 1 and 2 on the diagram), which included 100% of the total inertia (the inertia is the total variance of a dataset i.e. the trace of the correlation matrix). As to the contribution of individual groups of variables, a general equilibrium can be observed for axis 1 in a range that varies from 14.22% (Phenols) up to 18.12% (Others compounds) (Table 2). Different conclusions can be drawn regarding the contribution of each group of variables to axis 2. The contribution of sulfur compounds appears as to be the most statistically significant (21.46%). The contribution of aromatic hydrocarbons on the other hand, was low (6.14%): this is the least useful group of variables for the purpose of discriminating among the samples on the axis 2 of the MFA. The data provided by MFA was also subjected to further processing to determine how much each class of compounds (Fig. 2b) was useful for discriminating between samples. The same was done to determine the contribution of the individual compounds (Fig. 2c).

These data can be read in the same way as data in a normal PCA: the individual chemical classes correspond to the correlation coefficients between these variables and the factors. Compounds that significantly correlate (α = 0.05) to the two first dimensions are summarized in Table 3. Of these, 9 compounds were selected for their statistical relevance in the first dimension of MFA, while 8 compounds were chosen for being statistically relevant in the second dimension of MFA. Each truffles has six partial points corresponding to the chemical classes (Table 1). The length and the direction of the vectors are directly correlated to their significance within each population. Factorial axis 1 (57.64 % of the variance) clearly separated the truffles according to the harvest season, whereas the second axis (comprising the 42.36% of the variance) separated the winter samples according to geographic origin (Fig. 2b, individual factor map).

**Figure 3:**
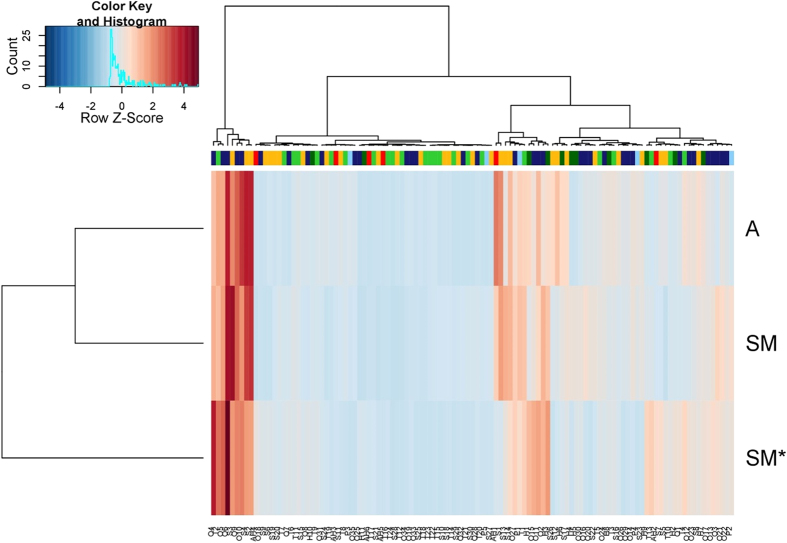
Heat-map based on the quantitative data for the three populations of analyzed samples. Signals were subdivided into classes based on chemical and biochemical features of the compounds. Darkgreen “H”, 1–11 hydrocarbons; red “AH” 1–6, aromatic hydrocarbons; goldenrod “S”, 1–30 sulphur compounds; midnightblue “O”, 1–35 others compounds; limegreen “T”, 1–24 terpenes; lightskyblue “P”, 1–5 phenols, respectively.

The third plot (“Correlation circle”) represents the normalized vectors of all quantitative variables. The angle between two arrows represents the correlation of the respective variables. There is no linear dependence if the angle is 90 degrees. In Fig. 2c, compounds belonging to the same class are arranged in a uniform manner in a correlation circle though it is not possible to identify any specific accumulation of compounds belonging to the same class. To further understand the differences and similarities between truffles, we next examined the compounds that were used to construct factor maps. The quantitative data are also depicted as a heat-map (Fig. 3) obtaining two dendrograms, one related to the samples and the other to the chemical structures. Both dendrograms were created independently of the heat map using correlation distance and the Ward method of agglomeration. The Ward method^24^ has a more statistical basis (Fig. 3). Using this method, the distance between groups is defined as the amount of information lost (or error created) by summarizing the objects into n clusters. The hierarchical clustering provided in the heat map confirmed the clustering obtained through MFA (data not shown). The two phylogenetic trees show that the "Marcia" sample is clearly differentiated from the other two samples (Fig. 3, left side of the diagram), while compounds within the same class cannot be grouped on the basis of their intensity signals as shown by the 4 groups of compounds obtained (Fig. 3, above diagram).

**Figure 4:**
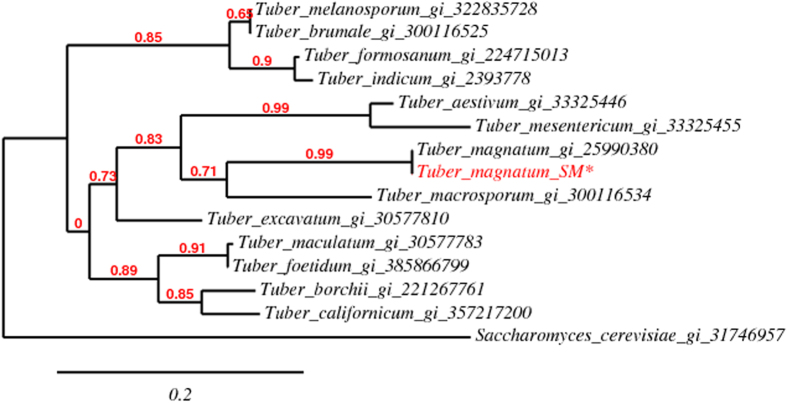
A phylogenetic tree shows the inferred evolutionary relationship between the SM* sample (obtained from Sanger sequencing) and other truffle species (sequences downloaded from NCBI (http://www.ncbi.nlm.nih.gov/). Each sequence is listed with by its own name and gene identificator. It can be noted that SM* appears to have the same sequence of the repository sequence of *T. magnatum* suggesting that both accessions belong to the same species. This tree was generated using *phylogeny.fr* (www.phylogeny.fr) in “One Click” mode^43^. The bar represents 0.2 nucleotide substitutions per position. *Saccharomyces cerevisiae* (gi 31746957)was used as outlier sample.

The occurrence of a reduced number of quantitatively relevant sulphur compounds can be explained on the base of the results obtained by sequencing the ITS regions of the "Marcia" sample (Fig. 4). The reverse and forward sequencing data of ITS1/ITS4 and ITS5/ITS6 fragments shows as the *T. magnatum* SM* present a 100% homology with the homologous deposited sequence of *T. magnatum*.

## Discussion

The first group to use PTR-MS to study *T. magnatum* aroma was Aprea’s group^18^. Following this initial publication, the use of PTR-MS gained acceptance as a reliable and rapid way to quantitatively analyze volatiles.

The VOCs profiles of *Tuber* spp. are highly complex and are far from being fully described. Many of the molecules identified in our experiments had previously been found in truffles collected in various european areas. However, 26% (29 out of 111) of them are being reported for the first time as volatiles produced in *Tuber* spp. (Table 1). Of these, 19 have been identified as BVOC (Biogenic Volatile Organic Compounds) in various other organisms. An additional 7, to our knowledge, have never been associated with any organisms, but have been found (by means of PTR-TOF-MS analysis) in food matrices^20^,^21^,^25^,^26^. The remaining 3 previously unidentified compounds in *Tuber* spp. (cyclopentenyl carbenium, 2-ethynylthiophene and 2,5-dimethylthiophene), have never before been cited as BVOC. Although knowledge of which VOCs are present in a given species is useful for identification purposes, it is difficult to distinguish between them based on single compounds. For this reason MFA analysis was used to grouping the VOCs into six most broad categories.

The resulting groups were analyzed to determine their usefulness as markers in distinguishing between samples (Table 3). With regard to axis 1, which is mostly related to the season, we see a general equilibrium in the contribution of each class whereas a different trend is visible along the axis 2, which is mostly related to the geographical origin of the samples. For axis 2, the contribution of each class of compounds is different, ranging from the 6.14% for aromatic hydrocarbons compounds (minimum value) to 21.46% for sulphur compounds (maximum value). This result may be related to the specific volatile profile of *T. magnatum* in which the sulphur compounds are considered by general consensus to be the main contributors to its unique flavor and are possibly the reason behind the price differences among truffles harvested from different regions.

Of the 17 compounds which best correlated with the two dimensions of the graphical display, 8 belongs to the group “Others”. This was the group that most strongly contributed to the first dimension of MFA (Fig. 2a–c) and includes 4 of the compounds that individually contributed the most to the first dimension (Pearson correlation >0.99, Table 3). Among these are aldehydic and ketonic compounds, which are not clearly assignable to any well-established metabolic pathway. One of these compounds was 2-methyl-1-propanol (previously reported in other species of truffle^12^,^27^) and another was acetaldehyde, whose presence in *T. magnatum* had previously been documented^18^. A quick examination of the second axis (axis 2) of MFA (42.36% of total variance) illustrates how the class “Others” contains the majority of the most representative compounds in this case too. One of these compounds was: i.e. the 2-methylbutanal, which had previously been found in *T. magnatum*^12^. A different scenario unfolds when considering the sulphur compounds which are often considered responsible for the distinctive aroma of the *T. magnatum* fruiting bodies. In this case only two compounds (one for each dimension of multifactorial analysis, Table 3) are significantly correlated with either of the two dimensions obtained by MFA analysis: diethanol sulfide and methylsulfanyl cyclopentane. Diethanol sulfide (negatively correlated to the first dimension, −1.000 of Pearson coefficient) and has previously been identified in *T. magnatum* as a compound able to distinguish between samples originating from different locations^5^. Methylsulfanyl cyclopentane negatively correlated to the second dimension (−1.000 of Pearson coefficient) and has previously been found in *Tuber borchii*^27^.

A study from 2008 had identified a series of characteristic compounds able to distinguish among samples of white truffle from seven Italian geographic areas^5^. In our experiments 6 of those signals (S3, S16, S22, S23, S27, T15, see Table 1 for more detail) were detected and we observed that, for these compounds there was a negative correlation of their Pearson coefficient related in the first dimension of MFA, whereas they generally appeared to correlate positively to second axis (**S3**, −0.535, 0.845; **S16**, −0.653, −0.758; **S22**, −0.788, 0.615; **S23**, −1.000, 0.027; **S27**, −0.136, 0.991; **T15**, −0.490, 0.872 for axis 1 and axis 2 respectively). These compounds did not distinguish between samples originating from our two different geographical locations though one of the compounds, S23, appeared to be specific to the Axis 1, and could possibly differentiate between summer and winter fruiting bodies.

Gioacchini^5^ found qualitative differences in sulfur compounds and terpenes among truffles originating from seven Italian areas, leading them to suggest that it might be possible to use intra-specific variation of VOC profiles to determine the area of origin of an unknown samples. Our data support this idea. In a series of detailed papers, Splivallo^4^,^17^ produced an in-depth description of truffles volatiles, concluding that since GC/MS instrument are less sensitive than the human nose, there is still room for improvement of the identification of truffle volatiles. By focusing their attention on *T. uncinatum,* Splivallo and coworkers found that C8-VOCs are major players in intraspecific aroma variability and they proved that 1-octen-3-ol does not occur exclusively in fully mature truffles. They also support the idea put forward by Gioacchini^5^ that if isoprenoids like cedrol and himachalene are to be used as marker for *T. magnatum* originating from Piedmont and Umbria, intra-specific genetic variability should be factored into the equation.

The aromatic profile of the summer sample “Marcia” (San Miniato Summer or SM^*^) is strikingly different from that of the other samples, San Miniato and Alba, harvested during the cold season. The data shows how SM*, harvested in summer, produces VOCs which distinguish from both A and SM, both harvested during the period of November-December. The cluster tree (Fig. 3, left side of the diagram) confirmed that the differences detected among the populations of samples are more significant when the fruiting body formation period is considered rather than the geographical origin. The second cluster tree (Fig. 3, above diagram) on the other hand, showed the presence of 4 groups of compounds represented by different signals belonging to different classes and assembled according to their intensity.

To our knowledge, these results include the first set of data published on a "Marcia" fruiting body belonging to the *T. magnatum* species, and reinforce the hypothesis that this species has two distinct biological phases for the production of the fruiting bodies. The fruiting bodies from the different phases present markedly different aromatic profiles.

It has been suggested that this kind of data might be useful for molecular barcoding in fungi because, using it, there is a good chance of successful identification of a very broad range of fungi^28^. The most clearly defined barcode gap would be between inter- and intraspecific variation. DNA barcoding is the use of a short gene sequence from a standardized region of the genome that can be used to help discover new species, as well as to characterize and distinguish between known species and assign unidentified individuals to species^29^. Results from the analysis of ITS5/ITS6 confirmed that the SM* sample belonged to the *T. magnatum* species, as shown in Fig. 4. Consequently, the differences observed between the summer and winter samples might be attributable to the environmental conditions, which vary considerably with the season (summer vs fall-winter) during the growth of fruiting bodies, which logically give rise to altered VOC's production.

In conclusion, although the greatest step forward on truffles volatiles was accomplished with the introduction of mass spectrometry, however the most recent progression is the birth of PTR-TOF-MS technology. Using this technique we were able to detect compounds at extremely low levels; the 111 compounds listed in this paper represent the higher number of VOCs reported in *T. magnatum* fruiting bodies, even though more work will be needed before a comprehensive picture is available.

Besides, the VOC analysis of the three different fruiting bodies made it possible not only to record the difference between the fruiting bodies of Alba and San Miniato, but also to distinguish between summer ("Marcia") and fall/winter production. VOC analysis proves that the "Marcia" stage of fruiting bodies, although analogous to the fruiting bodies collected during fall/winter develops specific metabolic characteristics as a result of the different season. To the best of author’s knowledge, this has been reported for the first time. It seems that for each season, the resulting truffles are, at least metabolically, quite distinct. On the other hand the fruiting bodies that grow during the summer are not deep in the soil as the winter ones; therefore they grow much faster and rapidly they rot. Consequently their entire metabolism, including the formation of VOCs, is different when compared with the winter fruiting bodies.

Finally, adequate description of truffle aroma requires the use of sophisticated tools due to its complexity, including an accurate in-depth statistical analysis of the data as was done in several figures of this paper. The limited data already collected by various scientists should be assembled with other new results as they become available. Such a methodology will allow for significant advances in knowledge of truffle VOC biology through the implementation of statistical analysis.

## Methods

### Fruiting bodies and PTR-TOF-MS analysis

VOCs emitted from samples were collected over three harvest seasons (2011–2013), from Piedmont (Alba, A) during the winter and from Tuscany (San Miniato) during winter (SM) and summer (SM*), “Marcia”. For each sample, three carpophores of about 10–15 g were collected and stored at 4 °C in glass vials and analysed within 24 h. Volatiles were analysed with a PTR-TOF-MS 8000 (IoniconAnalytik GmbH, Innsbruck, Austria) using H_3_O^+^ as reagent ion for the proton transfer reaction. The reaction takes place between H_3_O^+^ions and all the biogenic VOCs having a proton affinity higher than that of water (165.2 kcal mol^−1^). The separation of the resulting single ions depends on their mass to charge (*m/z*) ratio. The reaction takes place in a reaction chamber (Drift tube) under controlled conditions of applied voltage (set at 600 V), temperature (at 110 °C), and pressure (at 2.25 mbar). Compounds such as 1,4 dichlorobenzene (m/z = 146.976) and 1,2,3 trichlorobenzene (m/z = 180.937) were continuously used, together with other known low mass ions, for a precise conversion of ‘‘time-of-flight’’ into ‘‘mass-to-charge’’ ratio (*m/z*) in order to assign the exact mass scale and the sum formula of all ions during VOC analysis^23^,^30^. For each sample, about 10 grams of material were placed in a glass jar and covered with a special lid that allowed Teflon connection to a zero-air generator (inlet) and to the PTR-TOF-MS system (outlet). The head space was then measured by direct injection into the PTR-TOF-MS drift tube inlet for 150 seconds, after 10 minutes of exposure. Preliminary measurements on an empty jars were run before every experiment and used for background subtraction. All mass spectra up to *m/z* = 250 were simultaneously detected and recorded with 1 s as the integration time. Internal calibration was based on *m/z* = 21.0202 (H_3_^18^O^+^), *m/z* = 29.9974 (NO^+^), and *m*/*z* = 59.0491 (C_3_H_6_O^+^). For a more detailed explanation see references^23^,^30^. Data obtained by PTR-TOF analysis were processed as described in reference^31^. Briefly, raw spectra data (count rate of the analytes recorded were expressed in number of counts per second, cps) were acquired with TOFDaq software (TOFwerk AG, Switzerland) using a dead time of 20 ns for the Poisson correction and peak extraction followed the methodology described in reference^31^, employing a modified Gaussian peak shape. For peak quantification the resulting data were corrected according to the duty cycle and the signals were normalized to the primary ion signal (cps to ncps) as described in reference^32^. For each sample, the average data resulting from 20 consecutive seconds of measurement were extracted 3 minutes after the beginning of the experiment. All spectra were corrected for count losses due to the detector dead time, applying Poisson correction in the DAQ settings of TOFDAQ configuration options. External calibration was automatically done by the acquisition program and it achieved a mass accuracy of 0.001Th for the considered mass range, which was in most cases sufficient for formula identification.

### Statistical analysis

To identify relationships among the samples (Alba, A; San Miniato winter, SM; San Miniato summer SM*) based on data obtained from PTR-TOF-MS, multiple factorial analyses (MFA) was used^33^. MFA was performed in two steps. Firstly, a principal component analysis (PCA) was computed on each data set, which was then “normalized” by dividing all its elements, by the square root of the first eigenvalue obtained from of its PCA. Then, the normalized data sets are merged to form a single matrix and a global PCA is performed on this matrix. The individual data sets are then projected onto the global analysis to analyze communalities and discrepancies. Volatile compounds significantly contributing to MFA dimensions were used to explain differences among truffles (normal law adjustment test on compounds correlation coefficients, α = 0.05). A hierarchical clustering on principal components (HCPC) was performed to confirm the product groups observed graphically^34^. Heat maps method were used for visualizing complex data sets organized as matrices. A heat map does two things to a matrix. First, it reorders the rows and columns so that rows (and columns) with similar profiles are closer to one another, rendering them to be more visible to the eye. Second, each entry in the data matrix is displayed as a color, making it possible to view the patterns graphically. The dendrograms were created using correlation-based distances and the Ward method of agglomeration was used in the present analysis^35^. All computations were performed with R 3.0.3^36^ language and environment and R packages *FactoMineR*^37^, and *gplots*^38^ were used.

### PCR analysis for species identification

Total genomic DNA was extracted from a sample named as “Marcia” using the CTAB extraction method^39^,^40^ with minor modifications. Next, the ITS region was amplified with the ITS5/ITS6 pair of primers^41^ using a Biorad MyCycler system in a 25 μl of mixture solution containing 100 ng of DNA from fruiting bodies. Amplification was performed using the following protocol to get each sequence. PCR amplification with the pair of primers ITS5/ITS6 was carried using the method described in reference^41^. Electrophoresis on agarose gel (2 μl of PCR mixture, 2% agarose gel) with ethidium bromide staining confirmed that the PCR products were of the predicted size ITS5/ITS6 (600–650 bp). The amplicons were purified trough Wizard SV Gel and PCR Clean-Up System Kit (Promega) and then sequenced (BMR Genomics, Padova Italy) to get their relative sequences.

The sequences thus obtained were inserted into a multiple sequence alignment program, using the MUSCLE alignment algorithm^42^. A neighbor-joining tree was constructed based on maximum likelihood (PhyML) using the web resource available on the *phylogeny.fr* website (http://www.phylogeny.fr), an high performance platform designed to perform phylogenetic analysis based on a multiple alignment^43^. The phylogenetic tree constructed using this data helped define the species to which the summer fruiting bodies belong.

## Additional Information

**How to cite this article**: Federico, V. *et al*. Volatile organic compounds in truffle (*Tuber magnatum* Pico): comparison of samples from different regions of Italy and from different seasons. *Sci. Rep*. **5**, 12629; doi: 10.1038/srep12629 (2015).

## Figures and Tables

**Table 1:** Compounds identified through PTR-Analysis.

Compound s^a^	Measured, m/z^b^	Protonated chemical formula^c^	Chemical and functional grouping c^d^	Tentative of identifications^e^	Theoretical, m/z^f^	Compound classification^g^	PTR-TOF citations^h^	*Tuber magnatum* citations^i^	*Tuber*spp. Citations^l^	bVOCs citations^m^
1	27.025	C_2_H_3_+	H1	Acetylene	27.0229	O				46
2	29.039	C_2_H_5_+	H2	Alkyl fragment (ethanol)	29.0386	O	21			
3	31.042	CH_3_O+	O1	Formaldheyde	31.0178	X				46
4	33.033	CH_5_O+	O2	Methanol	33.0335	X	21,26	18	27	
5	34.995	H_3_S+	S1	Hydrogen sulfide	34.9949	X	21			44,51
6	41.039	C_3_H_5_ +	H3	Alkyl fragment	41.0385	X	20,21,26			
7	42.034	C_2_H_4_N+	O3	Acetonitrile	42.0338	X	21,26			
8	43.018	C_2_H_3_O+	O4	Alkyl fragment (ethenone)	43.0178	X	26			
9	43.054	C_3_H_7_+	H4	Alkyl fragment (propene)	43.0542	X	26			46
10	45.033	C_2_H_5_O+	O5	Acetaldehyde	45.0334	X	21,26	18	12,14,15,46,47	
11	46.990	CH_3_S+	S2	Methanethial	46.9949	O				48
12	47.013	CH_3_O_2_+	O6	Formic acid	47.0128	O	21,26			46
13	47.049	C_2_H_7_O+	O7	Ethanol	47.0491	X	21,26	10,12,18,49		14,46,47,50
14	49.011	CH_5_S+	S3	Methanethiol	49.0106	X	21,26	5,10,18		46,47,51
15	53.038	C_4_H_5_+	H5	Cyclobutadiene	53.0385	O				52,53
16	55.054	C_4_H_7_+	H6	Alkyl fragment	55.0542	X	26			
17	57.034	C_3_H_5_O+	O8	2-Propenal (Acrolein)	57.0334	O	21			54
18	57.068	C_4_H_9_+	H7	1-Butene (alkyl fragment)	57.0699	X	21			55
19	59.049	C_3_H_7_O+	O9	2-Propanone (acetone)	59.0491	X	21,26	10,12	14,15	46,47,56,57
20	61.028	C_2_H_5_O_2_ +	O10	Acetic acid	61.0284	X	21,26	49	12,15	46,47,58,59,60
21	63.026	C_2_H_7_S+	S4	Dimethyl sulfide	63.0262	X	21,26	5,10,12,18,49	14,15,16,17	46,51
22	65.023	CH_5_O_3_+	O11	Methanetriol	65.0233	O				61
23	67.054	C_5_H_7_+	H8	3-Penten-1-yne	67.0542	X			62	
24	68.05	C_4_H_6_N+	O12	Pyrrole	68.0494	X	20	10,11		
25	69.033	C_4_H_5_O+	T1	Furan	69.0336	X	20		63	
26	69.070	C_5_H_9_+	T2	2-Methyl-1,3- butadiene (isoprene)	69.0698	X	21		12	51,56
27	71.049	C_4_H_7_O+	O13	3-Buten-2-one	71.0491	X		10		
28	71.086	C_5_H_11_+	H9	Alkyl fragment (several compounds)	71.0855	O	20,26			
29	73.03	C_3_H_5_O_2_+	O14	Acrylic Acid	73.0284	X			15	
30	73.065	C_4_H_9_O+	O15	2-Butanone	73.0648	X	21,26		12,14,15,17,27	64
31	74.061	C_3_H_8_NO+	O16	Dimethylformamide	74.0610	O				65
32	75.044	C_3_H_7_O_2_+	O17	Methyl acetate	75.0441	X	21		14	
33	75.080	C_4_H_11_O+	O18	2-Methyl-1-propanol (isobutanol)	75.0804	X			12,27	46,51,56,60,64
34	77.01	C_3_H_9_S+	S5	1-Propanethiol	77.0055	O				55
35	78.046	C_2_H_6_OS+	S6	(Methylsulfinyl)methanide	78.0133	O				45
36	79.021	C_2_H_7_OS+	S7	Dimethyl sulfoxide (Sulfinylbismethane)	79.0212	X		18		
37	80.049	C_5_H_6_N+	O19	Pyridine	80.0494	X	20		27	
38	81.000	CH_5_S_2_+	S8	Bis(methylthio) methane	80.9827	X		10		
39	81.069	C_6_H_9_+	H10	Alkyl fragment (hexenals/hexenols/terpenoids)	81.0699	X	21,25,26			
40	82.06	C_6_H_10_+	H11	Cyclopentenyl carbenium	82.0777	#				
41	83.049	C_5_H_7_O+	T3	2-Methylfuran	83.0491	X	20	10	12	51
42	85.029	C_4_H_5_O_2_+	T4	5 h-Furan-2-one	85.0284	X			27	
43	86.018	C_4_H_6_S+	S9	Thiophenium	86.0184	O				66
44	87.043	C_4_H_7_O_2_+	O20	Butan-4-olide	87.0441	X			15	51
45	87.081	C_5_H_11_O+	O21	2-Methylbutanal	87.0804	X		12	15,17	46
46	88.030	C_4_H_8_S+	S10	3,4-Dihydro-2H-thiophene	88.0341	O				66
47	89.041	C_4_H_9_S+	S11	Allyl methyl sulfide	89.0419	X		5,9	12	
48	89.056	C_4_H_9_O_2_+	O22	3-Hydroxy-2-butanone (acetoin)	89.0597	X	21		15,27	46,50,51,60,67
49	91.055	C_4_H_11_O_2_+	O23	2-3-Butanediol	91.0753	X			12,27	46,51,67
50	93.035	C_3_H_9_OS+	S12	2-Methylmercaptoethanol	93.0368	O				51,68
51	93.068	C_7_H_9_+	AH1	Methylbenzene (toluene)	93.0698	X	21		12,15,69	55
52	95.010	C_2_H_7_O_2_S+	S13	Dimethyl sulfone	95.0035	X	20,26	10,18		
53	97.025	C_5_H_5_O_2_ +	T5	Furfural (2-Furancarboxaldehyde)	97.0284	X	25		15	46,67
54	97.065	C_6_H_9_O+	T6	2,4-Dimethylfuran	97.0647	X	20	10		
55	99.044	C_5_H_7_O_2_+	T7	4-Methyl-5 h-furan-2-one	99.080	X	20		27	
56	101.045	C_5_H_9_S+	S14	2-Methyl-4,5-dihydrothiophene	101.0419	X	20		13,27	
57	101.060	C_5_H_9_O_2_ +	O24	2,3-Pentanedione	101.0597	X	21,26	10		
58	103.050	C_8_H_7_+	AH2	Ethynylbenzene	103.0543	X			12,62	
59	103.076	C_5_H_11_O_2_+	O25	4-Hydroxy-3-methyl-2-butanone	103.0754	X			14	
60	105.037	C_4_H_9_OS+	S15	Methional	105.0368	X		5	16,27	51
61	105.060	C_4_H_9_O_3_+	O26	4-Hydroxybutanoic acid	105.0546	X		10		
62	106.995	C_3_H_7_O_2_S+	S16	2-Methylthioacetic acid	107.0161	X		5		
63	107.086	C_8_H_1 1_ +	AH3	1,3-Dimethylbenzene terpenes fragment	107.0855	X	21		15	
64	109.010	C_6_H_5_S+	S17	2-Ethynylthiophene	109.0106	#				
65	109.065	C_7_H_9_O+	P1	Anisole	109.0647	X			12,15	
66	111.044	C_6_H_7_O_2_+	T8	2-Acetylfuran	111.0431	O	20			51,70
67	111.104	C_8_H_15_+	T9	4-Methyl-1,3-heptadiene	111.1168	X			27	
68	113.040	C_6_H_9_S	S18	2,5-Dimethylthiophene	113.0419	#				
69	113.100	C_7_H_13_O+	T10	2-Heptenal	113.0960	X			15	
70	115.020	C_5_H_7_OS+	S19	2-Methyl-3-furanthiol	115.0212	X			16	
71	115.075	C_6_H_11_O_2_+	P2	3,5-Dimethyldiidro-2(3 h)-furanone	115.0746	X	20	10		51
72	117.078	C_6_H_13_S+	S20	Cyclopentyl-1-thiaethane (methylsulfanyl cyclopentane)	117.0732	X			27	
73	119.06	C_6_H_15_S+	S21	1-(Methylthio)pentane	119.0385	X		5		
74	121.065	C_5_H_13_OS+	S22	2-Hydroxyethyl propyl sulfide	121.0681	X		5		
75	121.101	C_9_H_1 3_+	AH4	1,2,4-Trimethylbenzene	121.0647	X			15	
76	123.045	C_4_H_11_O_2_S+	S23	Diethanol sulfide	123.0474	X		5		
77	123.080	C_8_H_11_O+	P3	1-Methoxy-3-methylbenzene	123.0804	X			12,14,15,27	
78	125.010	C_3_H_9_OS_2_+	S24	(Methylsulfinyl)(methylthio)methane	125.0089	X		5		
79	125.096	C_8_H_13_O+	T11	2-Butylfuran	125.0961	X	26	10		
80	127.035	C_6_H_7_O_3_+	T12	Methyl 2-Furancarboxylate	127.0389	O				51,71,72
81	127.112	C_8_H_15_O+	O27	1-Octen-3-one	127.1117	X	26	10		
82	129.070	C_10_H_9_+	AH5	Naphthalene	129.0698	X			15	50
83	129.127	C_8_H_17_O+	T13	c8 aldehydes and ketones (2-octanone)	129.1273	X	26	10		
84	131.085	C_7_H_15_S+	S25	Methylsulfanylcyclohexane	131.0888	X			27	
85	131.107	C_7_H_15_O_2_+	O28	Ethyl 2- methylbutanoate (Ethyl 2-methylbutyrate)	131.1066	X			12,14,27	
86	133.101	C_10_H_13_+	T14	p-Cymenene	133.1011	X		10		
87	135.117	C_10_H_15_+	T15	p-Cymene	135.1168	X		5,18		
88	137.134	C_10_H_17_+	T16	Terpenes (Limonene)	137.1325	X	26	5,10,18,49		
89	139.148	C_9_H_15_O+	T17	2-Pentylfuran	139.1117	X		10	12,15	50
90	141.130	C_9_H_17_O+	T18	3-Nonen-2-one (several compounds)	141.1273	X		10		
91	143.144	C_9_H_19_O+	O29	Nonanal	143.1430	X	20,26	10		73
92	143.107	C_8_H_15_O_2_+	O30	2,3-Octanedione	143.066	X	26,30		15	
93	145.076	C_10_H_9_O+	T19	3-Phenyl-furan	145.043	X			27	
94	147.120	C_8_H_19_S+	S26	2-Ethyl-1-hexanethiol	147.1201	X		5		
95	149.130	C_11_H_17_+	AH6	(1-Ethylpropyl)benzene	149.1324	X			27	
96	151.065	C_6_H_15_S_2_+	S27	Methyl pentyl disulfide	151.0609	X		5		
97	153.130	C_10_H_17_O+	T20	2-Methyl-5-(1-methylethenyl)-2-cyclohexen-1-ol (Carveol)	153.1273	X	25	5		
98	155.010	C_4_H_11_S_3_+	S28	Diethyl trisulfide	155.0017	X		5		
99	157.159	C_10_H_21_O+	O31	Decanal	157.1586	X	22	10		
100	159.140	C_9_H_19_O_2_+	O32	2-Methylbutyl 2-methylpropanoate	159.1379	X			12,27	
101	161.155	C_9_H_21_O_2+_	O33	1,9-Nonanediol	161.1536	O				55,74
102	163.075	C_11_H_1 5_O+	O34	3-Methyl-2-(penta-2,4-dienyl)cyclopent-2-enone	163.0753	X			75	
103	167.140	C_11_H_1 9_O+	T21	2-Heptylfuran	167.1430	X		10		50
104	169.085	C_9_H_13_O_3_+	P4	1,2,4-Trimethoxybenzene	169.0859	O			12	
105	171.080	C_12_H_1 1_ O+	O35	2-Undecanone	171.0804	X		10		
106	175.010	C_6_H_7_O_4_S+	S29	4-Hydroxybenzenesulfonic acid	175.0059	X		5		
107	187.11	C_13_H_1 5_O+	P5	2-Hydroxy-4-isopropylnaphthalene	187.1117	X			27	
108	195.180	C_13_H_23_O+	T22	6,10-Dimethyl-5,9-undecadien-2-one (geranylacetone)	195.1743	X			17,75,76	
109	197.050	C_7_H_17_S_3_+	S30	Methyl(methylthio)methyl Disulfide	197.0486	X		5		77
110	205.195	C_15_H_25_+	T23	Sesquiterpenes	205.195	X	25	5		
111	223.200	C_15_H_27_O+	T24	Cedrol	223.2056	X		5		

^a^Compound rank.

^b^Mass to charge ratio measured by the Mass Spectrometer.

^c^Compound’s Chemical formula (H^+^ added by protonation).

^d^Compound classification based on their chemical and biochemical properties: AH aromatic hydrocarbon, H hydrocarbon, P phenol, S sulfur compound, T terpene, O others.

^e^Putative identifications according to spectral properties.

^f^Theoretical mass to charge ratio found in literature or PTR-TOF-MS manual.

^g^Compounds were marked related to their bibliography, X = Previously published in *Tuber magnatum* or *Tuber* spp., O = Similar signal properties to previously published compounds, # = Signals not previously reported.

^h^PTR-TOF-MS articles where molecule was reported.

^i^*T. magnatum* citations of molecule.

^l^*Tuber* spp. citation of that article.

^m^biological Volatile Organic Compounds (bVOC) previously reported.

**Table 2:** Compounds classes and their relative contribution to perform MFA dimensions.

Compound class^a^	Dimension 1	Dimension 2
*Other compounds*	18.12	17.89
*Hydrocarbons*	17.99	16.42
*Aromatic hydrocarbons*	17.75	6.14
*Sulphur compounds*	17.65	21.46
*Terpene compounds*	14.27	18.29
*Phenol compounds*	14.22	19.80

Each dimension of a multivariate analysis can be described by the variables which participate to the construction of the factorial axes.

^a^Compound classes are sorted according to their relative contribution of dimension 1.

**Table 3:** Compounds significantly correlated to first and second dimensions of the multiple factor analysis (MFA).

Tentative of Identification	Chemical Protonated Formula	First dimension	Pearson correlation coefficient	Tentative of Identifications	Chemical Protonated Formula	Second dimension	Pearson correlation coefficient
***Alkyl fragment (ethenone)***	C_2_H_3_O+	*O4*	*1.000*	***2-Hydroxy-4-isopropylnaphthalene***	C_13_H_15_O+	*P5*	*1.000*
***Alkyl fragment***	C_5_H_11_+	*H9*	*1.000*	***2,4-Dimethylfuran***	C_6_H_9_O+	*T6*	*1.000*
***2-Heptenal***	C_7_H_13_O+	*T10*	*1.000*	***Cyclopentyl-1-thiaethane***	C_6_H_13_S+	*S20*	*1.000*
***2-Methyl-1-propanol***	C_4_H_11_O+	*O18*	*1.000*	***2-Methyl-5-(1-methylethenyl)-2-cyclohexen-1-ol (Carveol)***	C_10_H_17_O+	*T20*	*0.998*
***2-Pentylfuran***	C_9_H_15_O+	*T17*	*1.000*	***2,3-Octanedione***	C_8_H_15_O_2_+	*O30*	*0.998*
***Acetaldehyde***	C_2_H_5_O+	*O5*	*1.000*	***Ethyl 2- methylbutanoate ((Ethyl 2-methylbutyrate)***	C_7_H_15_O_2_+	*O28*	*0.997*
***c8 aldehydes and ketones***	C_8_H_17_O+	*T13*	*0.998*	***2-Methylbutanal***	C_5_H_11_O+	*O21*	*0.997*
***Diethanol sulfide***	C_4_H_11_O_2_S+	*S23*	*−1.000*	***Pyridine***	C_5_H_6_N+	*O19*	*−0.997*
***Acrylic Acid***	C_3_H_5_O_2_+	*O14*	*−1.000*				

The selection of significant compounds was done based on their correlation coefficients (α = 0.05) and sorted by Pearson correlation coefficient.
