# Corrigendum: Volatile organic compounds in truffle (*Tuber magnatum* Pico): comparison of samples from different regions of Italy and from different seasons

**DOI:** 10.1038/srep24961

**Published:** 2016-04-29

**Authors:** Federico Vita, Cosimo Taiti, Antonio Pompeiano, Nadia Bazihizina, Valentina Lucarotti, Stefano Mancuso, Amedeo Alpi

Scientific Reports 5: Article number: 1262910.1038/srep12629; Published online: 07302015; Updated: 04292016

The original version of this Article contained errors in the spelling of the authors Federico Vita, Cosimo Taiti, Antonio Pompeiano, Nadia Bazihizina, Valentina Lucarotti, Stefano Mancuso and Amedeo Alpi which were incorrectly given as Vita Federico, Taiti Cosimo, Pompeiano Antonio, Bazihizina Nadia, Lucarotti Valentina, Mancuso Stefano & Alpi Amedeo respectively.

 The Author Contributions Statement,

V.F., A.A. and L.V. designed the research. V.F., T.C. and P.A. performed the experiments and analysed the data. V.F., A.A., M.S. and B.N. organized and drafted the paper with all authors contributing to the discussion of the data and to the writing.

now reads:

F.V., A.A. and V.L. designed the research. F.V., C.T. and A.P. performed the experiments and analysed the data. F.V., A.A., S.M. and N.B. organized and drafted the paper with all authors contributing to the discussion of the data and to the writing.

In addition, the ‘How to cite this article’ section quoted an incorrect abbreviation for Federico Vita.

These errors have now been corrected in the PDF and HTML versions of the Article.

